# Association Between Tobacco and Periodontal Disease in Latin America from 2000 to 2024: Cross-Sectional Analysis of Global Burden of Disease Study

**DOI:** 10.3390/jcm14103549

**Published:** 2025-05-19

**Authors:** Brenda Herrera-Serna, Olga López-Soto, Héctor Fuentes-Barría, Raúl Aguilera-Eguía, Lissé Angarita-Davila, Diana Rojas-Gómez

**Affiliations:** 1 Facultad de Salud, Departamento de Salud Oral, Universidad Autónoma de Manizales, Caldas 170008, Colombia; bherrera@autonoma.edu.co (B.H.-S.); sonrie@autonoma.edu.co (O.L.-S.); 2 Vicerrectoría de Investigación e Innovación, Universidad Arturo Prat, Iquique 1110939, Chile; 3 Departamento de Salud Pública, Facultad de Medicina, Universidad Católica de la Santísima Concepción, Concepción 3349001, Chile; raguilerae@ucsc.cl; 4 Escuela de Nutrición y Dietética, Facultad de Medicina, Universidad Andres Bello, Concepción 3349001, Chile; lisse.angarita@unab.cl; 5 Escuela de Nutrición y Dietética, Facultad de Medicina, Universidad Andres Bello, Santiago 7550000, Chile; diana.rojas@unab.cl

**Keywords:** tobacco products, tobacco use, periodontal diseases, Latin America

## Abstract

**Objectives:** This study aims to examine the ecological-level association between active and passive tobacco use and periodontal disease in Latin America from 2000 to 2024. **Methods:** A cross-sectional ecological study was conducted using secondary data from the Global Burden of Disease Study. Data from 20 Latin American countries were analyzed, stratified by country, sex, and age group. Multiple regression models were used to assess the relationship between tobacco consumption and periodontal disease prevalence, adjusted for age and sex. **Results:** The prevalence of periodontal disease was high in both sexes, particularly among individuals older than 55 years. The countries with the highest age-standardized rates were Colombia, Panama, and Costa Rica, with nearly 35,000 cases per 100,000 population. Regression models indicated that passive tobacco exposure explained 90.4% of the variability in women (R^2^ = 0.9041) and 92.5% in men (R^2^ = 0.9253). Active tobacco use showed weaker associations, with R^2^ values of 0.3721 in women and 0.4601 in men. Passive exposure demonstrated better predictive accuracy, with lower Root MSE values (3192.8 and 3261.7). **Conclusions:** There is a significant ecological-level association between tobacco use and periodontal disease in Latin America, particularly for passive exposure. These findings highlight the need to strengthen tobacco control policies and preventive strategies targeting environmental exposure. However, due to the ecological nature of the study, these associations do not imply causality at the individual level. Longitudinal studies with individual-level data are needed to explore the underlying biological and contextual factors.

## 1. Introduction

Periodontal disease is the most studied oral condition in relation to smoking, with its clinical effects and biological mechanisms increasing smokers’ susceptibility to developing periodontitis [1,2,3]. Recent evidence indicates that smokers have a higher prevalence, greater severity, and more rapid progression of periodontal disease compared to non-smokers and even former smokers. These negative effects significantly compromise the success of periodontal treatments, both conventional and surgical or regenerative [4,5].

In particular, heavy smokers tend to develop more aggressive forms of periodontal disease and exhibit a less favorable response to therapeutic interventions [6]. This clinical progression is largely attributed to immunological alterations induced by smoking, such as neutrophil and monocyte dysfunction, changes in antibody production, and the release of inflammatory mediators; factors that collectively promote the proliferation of periodontal pathogens [7,8,9].

Regular tobacco use, in its various form’s (cigarettes, bidis, pipes, or cigars), has also been associated with an increased risk of dental and gingival pigmentation, tumor lesions, periodontal diseases, edentulism, peri-implant bone loss, and treatment failure with dental implants [10,11,12,13]. At the same time, the use of electronic cigarettes has increased in recent years, promoted as a less harmful alternative. However, these devices contain multiple toxic agents that make them a silent threat to both oral and systemic health [14].

Moreover, the use of smokeless tobacco, especially products like gutka, has grown, with components that have been associated with a higher periodontal risk due to their ability to induce inflammation and suppress the growth of gingival keratinocytes and periodontal fibroblasts [15,16]. On the other hand, smokeless nicotine products also negatively affect oral health by promoting gingival recession, the formation of periodontal pockets, and the accumulation of bacterial plaque, thus contributing to the development of periodontitis [17]. In this context, passive exposure to tobacco smoke represents a risk comparable to active smoking, with adverse effects on both the oral cavity and general health, highlighting the need to accurately evaluate both forms of tobacco exposure [18,19].

In Latin America, the epidemiological study of periodontal disease faces significant challenges. The scarcity of population-based studies with rigorous methodologies, the underestimation of structural variables such as socioeconomic level, and the fragmentation of available data complicate the establishment of solid associations with factors such as tobacco consumption. These limitations hinder the design and implementation of effective public policies to address this issue in the region [20,21].

For these reasons, this study aimed to determine the association between tobacco consumption, both active and passive, and periodontal disease in Latin America during the period between 2000 and 2024.

## 2. Materials and Methods

### 2.1. Design

This study adopted a cross-sectional ecological design, in accordance with the Strengthening the Reporting of Observational Studies in Epidemiology (STROBE) guidelines for observational studies [22] and the Guidelines for Accurate and Transparent Health Estimates Reporting (GATHER) for studies based on population health estimates [23]. Secondary data were obtained from the Global Burden of Disease Study (GBD), developed by the Institute for Health Metrics and Evaluation (IHME), which justified the joint application of both methodological guidelines [24].

The unit of analysis consisted of mixed population groups, stratified by country, sex, and age group, from 20 Latin American countries during the period 2000–2024. This study was approved by the Ethics Committee of the Autonomous University of Manizales (Protocol Code: 026/109; Approval Date: 18 November 2024), as part of the macroproject “Burden of Oral Diseases in Latin America”, in accordance with Resolution No. 8430 of 1993 from the Colombian Ministry of Health and the Declaration of Helsinki [25].

### 2.2. Context

This study included the general population of the following Latin American and Caribbean countries: Argentina, Bolivia, Brazil, Chile, Colombia, Costa Rica, Cuba, Ecuador, El Salvador, Guatemala, Haiti, Honduras, Mexico, Nicaragua, Panama, Paraguay, Peru, the Dominican Republic, Uruguay, and Venezuela. Associations between tobacco use (active and passive) and the prevalence of periodontal disease were analyzed.

### 2.3. Participants

Modeled data from the GBD were used for individuals of both sexes, aged 15 years and older, residing in the aforementioned countries. The estimates included the prevalence of periodontal disease and tobacco use, considering both active use (conventional cigarettes, electronic cigarettes, and smokeless tobacco) and passive exposure (secondhand smoke). Data were stratified by country, sex (male and female), and age group (<20 years, 21–54, 55–85, and >85 years), allowing for the calculation of aggregated estimates at the regional level.

### 2.4. Variables

Prevalence of periodontal disease: the GBD classifies severe periodontitis as a level 2 condition, clinically defined by a Community Periodontal Index (CPI) score of 4, clinical attachment loss (CAL) > 6 mm, and probing pocket depth (PPD) > 5 mm. This condition represents one of the leading causes of years lived with disability worldwide, although it is associated with a low disability weight, as symptoms such as gingival bleeding or halitosis do not always interfere with daily activities [26].

Prevalence of tobacco use: the GBD identifies tobacco use as a level 1 modifiable risk factor, categorized under addictive substances (level 2). The categories used included current smokers, former smokers, and individuals exposed to secondhand smoke. This approach positions tobacco use as one of the most significant preventable contributors to the global burden of disease [27].

### 2.5. Bias

The potential for ecological bias is acknowledged, as associations observed at the population group level do not necessarily reflect causal relationships at the individual level. Measurement bias may also be present, given that the data originate from diverse sources (e.g., population surveys and administrative records) with variable quality and accuracy [28].

### 2.6. Sample Size

As this was an ecological study based on secondary data, a traditional sample size calculation was not performed. All available observations from 2000 to 2024 for the selected countries were included. The estimates covered individuals aged 15 years and older, grouped by country, sex, and age. The unit of analysis was the aggregated population group rather than individuals, which is inherent to this study design.

### 2.7. Quantitative Variables

The main quantitative variables were the prevalence of periodontal disease and the prevalence of tobacco use (active and passive), expressed as age-standardized rates per 100,000 population. These variables were treated as continuous in multiple regression models. Assumptions of residual normality, homoscedasticity, and linearity were verified. No variable transformations were required, as the models met the expected visual and statistical fit criteria. To facilitate interpretation and explore potential differential effects, age categories were defined (<20, 21–54, 55–85, and >85 years), and analyses were stratified by sex and country.

### 2.8. Statistical Analysis

Data were analyzed using IBM SPSS Statistics, version 27. Variables were described using frequencies, percentages, and age-standardized prevalence rates per 100,000 population. To assess the association between the prevalence of periodontal disease and tobacco use (active and passive), multiple regression models were applied, adjusted for age and sex. The age categories used were: <20 years, 21–54 years, 55–85 years, and >85 years.

Given the heterogeneity in data quality across the 20 Latin American countries included, all GBD data underwent preprocessing steps involving standardization and harmonization procedures, as outlined by the GBD methodology. This included adjustments for under-reporting and differences in case definitions between countries, using established modeling tools such as DisMod-MR and CODEm to enhance comparability and consistency of estimates [29]. Furthermore, cross-validation techniques and uncertainty intervals provided by GBD were considered to evaluate the robustness and reliability of the modeled prevalence rates used in the analyses.

Independent variables included active tobacco use (cigarettes, electronic cigarettes, and smokeless tobacco) and passive exposure (environmental tobacco smoke). The adjusted coefficient of determination (adjusted R^2^) was reported as a measure of explained variability. Assumptions of residual normality and linear relationships between variables were assumed. Statistical significance was set at *p* < 0.05, with 95% confidence intervals.

## 3. Results

Data from 20 Latin American and Caribbean countries were analyzed, with observations aggregated by country, sex, and age group. The unit of analysis was the population group, with no relevant missing data for the main variables. A total of 12,000 observations per sex, country, year, and condition were included, corresponding to the available data for the period 2000–2021 and projections to 2024.

### 3.1. Descriptive Data

**Figure 1:**
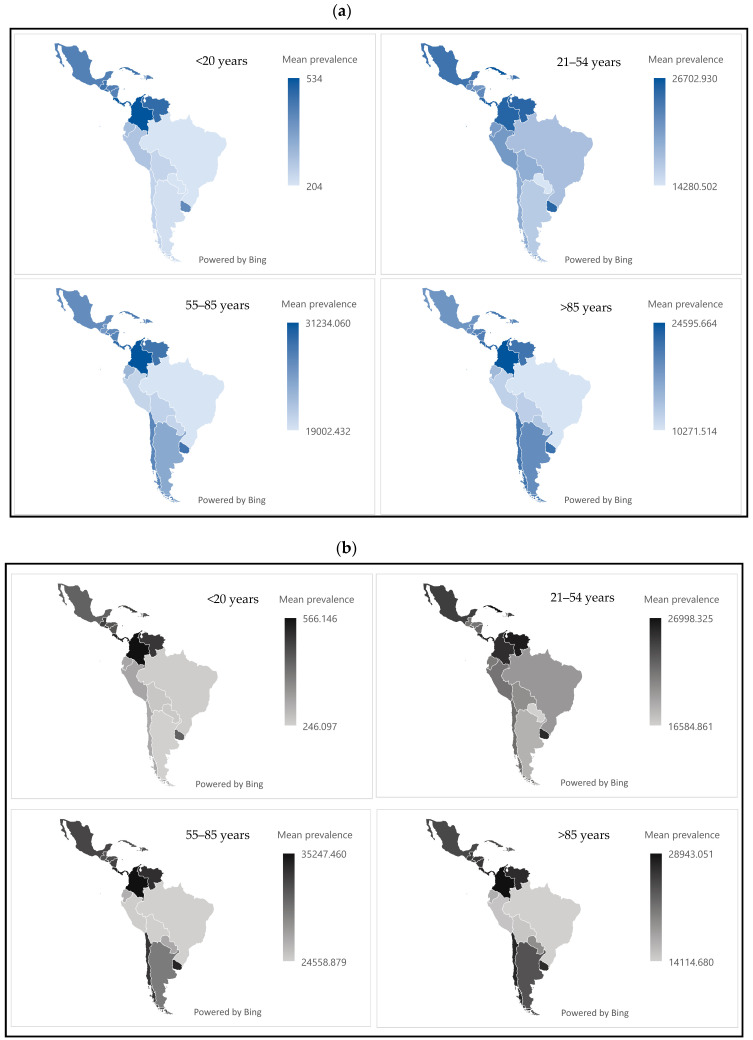
(**a**) Mean prevalence of periodontal disease in women by age group in Latin American and Caribbean countries between 2000 and 2024. (**b**) Mean prevalence of periodontal disease in men by age group in Latin American and Caribbean countries between 2000 and 2024. Source: Authors’ own elaboration.

Figure 1 presents the prevalence of periodontal disease by sex, age group, and country. In both sexes, the highest prevalence was observed in the 55–85 age group. The countries with the highest age-standardized rates were Colombia, Panama, and Costa Rica, with values close to 35,000 cases per 100,000 population.

### 3.2. Prevalence of Passive Tobacco Exposure

**Figure 2:**
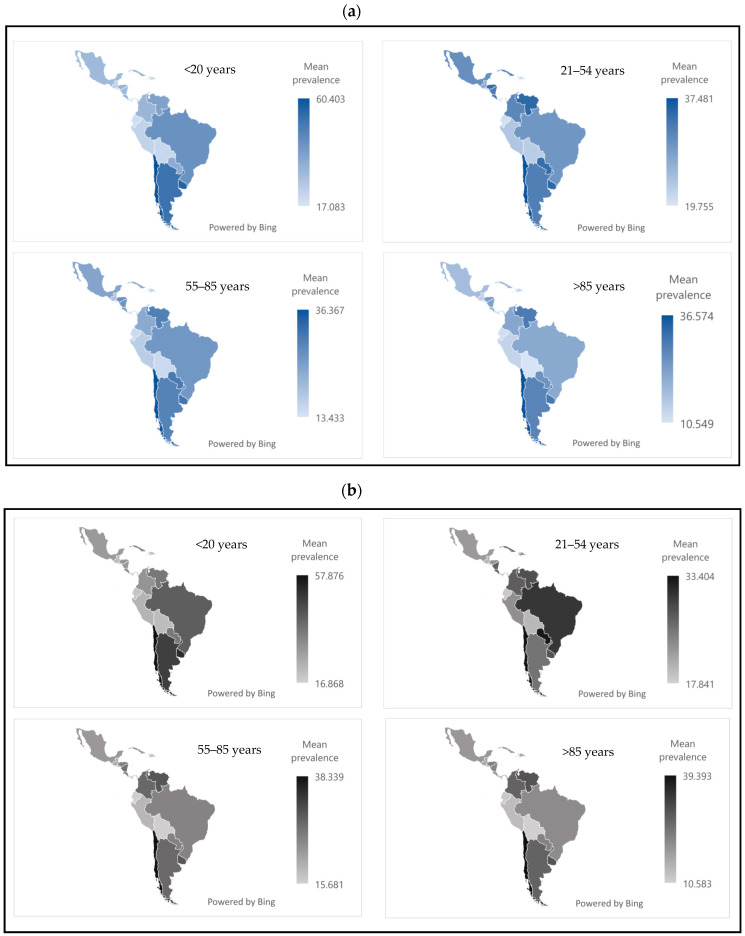
(**a**) Prevalence of passive tobacco exposure in women in Latin America and the Caribbean in 2024. (**b**) Prevalence of passive tobacco exposure in men in Latin America and the Caribbean in 2024. Source: Authors’ own elaboration.

Figure 2 shows the mean prevalence of passive tobacco exposure by sex, country, and age group. In both sexes, the <20 age group was the most affected. Chile, Uruguay, and Argentina reported the highest prevalence rates, ranging from approximately 50 to 60.

### 3.3. Prevalence of Active Tobacco Exposure

**Figure 3:**
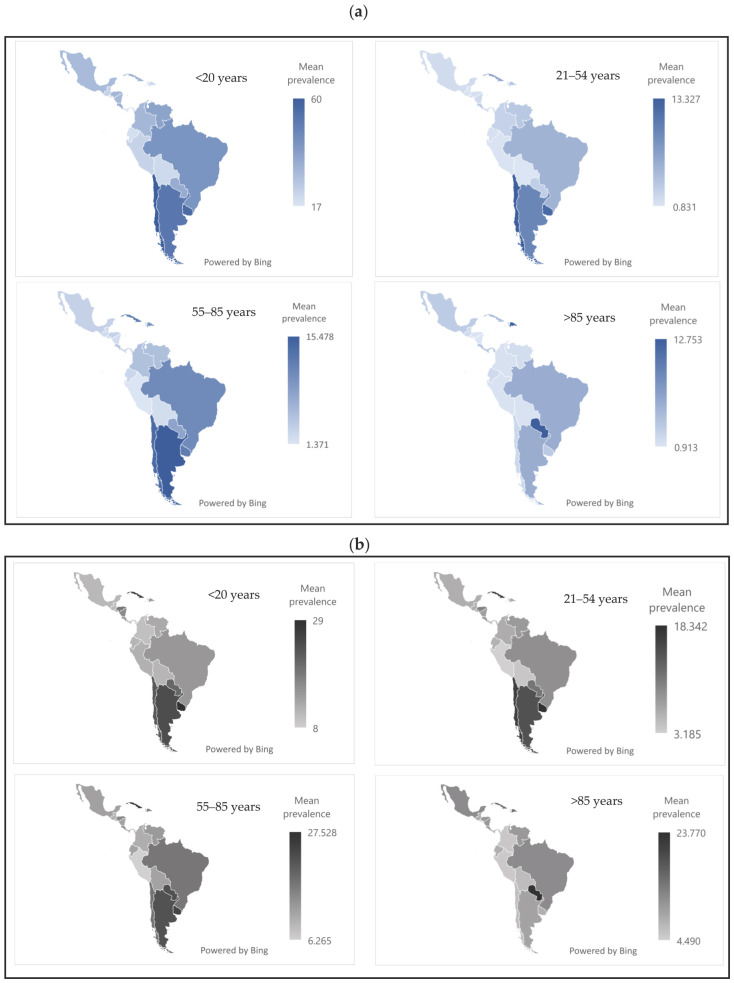
(**a**) Mean prevalence of active tobacco use in women in Latin America and the Caribbean in 2024. (**b**) Mean prevalence of active tobacco use in men in Latin America and the Caribbean in 2024. Source: Authors’ own elaboration.

Figure 3 shows that active tobacco use was most frequent in the 55–85 age group, in both sexes. The countries with the highest prevalence were Cuba, Uruguay, and Argentina, with values ranging from 12% to 25%.

### 3.4. Association Between Tobacco Use and Periodontal Disease

Table 1 presents the results of the multiple regression models, adjusted for age, that assessed the ecological-level association between the prevalence of periodontal disease and tobacco use (active and passive) by sex.

In women, at the ecological level, the model with passive tobacco exposure explained 90.4% of the variability (adjusted R^2^ = 0.9041), showing positive and statistically significant associations in the 21–54, 55–85, and ≥85 age groups. These results should not be interpreted as causal or applicable at the individual level. The model with active tobacco use presented an adjusted R^2^ of 0.3721, with a significant positive association in the 55–85 age group and a significant negative association in the ≥85 age group, again reflecting ecological-level trends rather than individual risk.

In men, at the ecological level, the model with passive tobacco exposure explained 92.5% of the variability (adjusted R^2^ = 0.9253), with significant positive associations from age 21 onward. However, despite the high R^2^ value, the strength and consistency of the regression coefficients should be interpreted with caution, as not all were statistically significant across age groups. In contrast, the model with active tobacco use had an adjusted R^2^ of 0.4601, with a significant association only in the 55–85 age group. As with the female data, these associations reflect population-level patterns and do not imply causality or direct individual-level effects.

The value of the Root MSE, included in all models, indicates the average prediction error of the model in the same units as the dependent variable. The lower values (3192.8 and 3261.7) correspond to the models with passive tobacco exposure, reinforcing their greater predictive accuracy within this ecological framework, not at the individual level.

These findings suggest, at the ecological level, that passive tobacco exposure is a more consistent and sensitive factor for explaining the prevalence of periodontal disease, compared to active tobacco use, especially in the mid-to-late stages of the life cycle. However, due to the ecological design, these associations should not be interpreted as evidence of causality or individual-level relationships.

## 4. Discussion

In the present study, a high prevalence of smoking was observed across all Latin American countries analyzed. Cigarette consumption is a widely recognized risk factor for the development of periodontal diseases. In this context, tobacco use trends in the region show notable heterogeneity: some countries, such as Mexico and Brazil, are at more recent stages of the smoking epidemic, while others, such as Argentina, Chile, Cuba, and Uruguay, have a more established epidemiological evolution.

This variability reflects both the degree of success and the differences in the design, implementation, and effectiveness of tobacco control policies adopted in each country. However, the threat of an increase in tobacco-related mortality persists, particularly in those contexts where smoking initiation occurred early, and control interventions have been late or insufficient [30]. This occurs despite the adoption of key MPOWER package measures (monitoring consumption, protection from smoke, offering help to quit smoking, warnings about the dangers of tobacco, banning advertising, and raising tobacco taxes), which, although they have contributed to reducing smoking in certain countries, still face significant implementation challenges, especially regarding the protection of the most vulnerable populations [31].

In this scenario, differences in tobacco use prevalence between men and women could be influenced by neurobiological mechanisms related to nicotine and dopamine [32]. Nicotine has the ability to increase the regulation of nicotinic acetylcholine receptors (nAChR), which has been linked to a faster release of dopamine in the dorsal striatum in women [33,34]. Additionally, it has been observed that alteration of nAChR in dopaminergic neurons can interfere with the behavioral effects of acute nicotine in men [32]; therefore, dopaminergic activity and nicotine-induced responses may be modulated by biological sex.

This study highlighted, through regression analysis, a significant association between periodontal disease and active tobacco use. While oral biofilm is recognized as the main etiological factor in periodontal diseases, it is widely acknowledged that its development and progression follow a multifactorial etiology, where environmental factors like smoking play a key modulatory role in prognosis and clinical evolution [35]. In this context, cytokines induced by bacteria, such as interleukin-8 (IL-8), promote leukocyte recruitment and contribute to gingival inflammation, while matrix metalloproteinase-8 (MMP-8) is responsible for collagen degradation and other components of the extracellular matrix, exacerbating the tissue destruction characteristic of periodontitis [36,37]. Likewise, it has been reported that low concentrations of cigarette extract can promote bacterial biofilm formation and trigger cellular stress reactions, with tobacco being a promoter of microbial dysbiosis in the oral cavity by modulating both bacterial metabolism and viability [18,38].

Consequently, many of the underlying effects of tobacco products on periodontal tissues can be attributed to a direct inhibition of gingival fibroblast and periodontal ligament function, where cellular viability is reduced in the presence of increasing concentrations of cigarette smoke extract and nicotine [39]. In this sense, the literature suggests that smoking increases the prevalence and quantity of certain oral bacterial strains [40]; thus, active smoking not only alters host responses including vascular function and neutrophil/monocyte activity but also increases the expression of adhesion molecules and antibodies associated with the release of inflammatory mediators, promoting the loss of alveolar bone homeostasis [41,42].

Regarding passive tobacco consumption, the present study found a possible association with periodontal disease in individuals aged 21 and older, up to 85 years. However, it should be noted that although the regression models were statistically significant, the coefficients for the predictor (passive exposure) did not reach significance in all groups, suggesting caution in interpreting this relationship.

In the present study, a significant negative association was observed between active tobacco use and periodontal disease prevalence in the age group ≥85 years. this may be explained by the “survivor bias” or “healthy survivor effect”, where more susceptible individuals have prematurely died, leaving a relatively healthier elderly cohort. Additionally, smoking rates typically decrease with advancing age, and other age-related factors such as systemic diseases or limited dental care access may impact periodontal disease assessment in this group [43,44]. Thus, this negative association does not imply a protective effect of smoking but reflects ecological study limitations and the particularities of the older population. Caution is warranted, and longitudinal individual-level studies are needed to further explore this finding.

An important limitation of this ecological study is the absence of adjustment for critical confounding variables such as socioeconomic status, education, and healthcare access [45]. These unmeasured factors can substantially influence both tobacco exposure and periodontal disease prevalence, potentially biasing the observed associations and limiting the explanatory power of regression models [46,47,48]. For example, individuals with lower socioeconomic status often have higher tobacco use rates and poorer oral health outcomes due to reduced access to preventive care and health education [45]. Therefore, the results should be interpreted with caution, recognizing that the associations observed at the population level may be influenced by these unmeasured confounders. Future studies incorporating individual-level data and these important variables are necessary to better understand the complex relationships between tobacco exposure and periodontal health in Latin America [49,50].

This situation underscores the need for public policies to focus not only on reducing active consumption but also on expanding protection against environmental tobacco smoke exposure [20].

In this sense, some reports indicate that passive smokers may be more likely to develop periodontitis compared to those who are not exposed [42]. Thus, exposure to environmental tobacco smoke may be a relevant modulator of infection rates by periodontopathogens like Treponema denticola and Porphyromonas gingivalis, whose action on IL-8 and salivary MMP-8 could distinguish clinical findings in both passive and active smokers. However, given the broad confidence intervals and marginal *p*-values observed, this association should be approached with caution and requires further validation through longitudinal studies with individual data [18].

## 5. Strengths and Limitations

One of the main strengths of this study was the joint application of the STROBE and GATHER guidelines, which enabled a rigorous, transparent, and structured presentation of methods, results, and analytical assumptions. The integration of STROBE facilitated the systematic reporting of an observational ecological cross-sectional design, while GATHER ensured clear and detailed documentation of sources, models, and population estimates. This combined methodological approach enhances the reproducibility and analytical traceability, both essential elements in research based on modeled secondary data.

Additionally, the use of the GBD as an information source, a well-established, validated, and widely used database in comparative disease burden studies, provided a solid foundation for the study. Its hierarchical structure and standardization enabled comparisons between countries and demographic subgroups over an extended period (2000–2024), which adds robustness to the regional findings.

However, there are relevant limitations that should be considered. First, the ecological design with aggregated data prevents the establishment of causal relationships or the direct extrapolation of findings to the individual level, exposing the results to the risk of ecological fallacy.

Second, although the GBD employs advanced statistical models to adjust and estimate data in contexts with incomplete information, the estimates remain subject to methodological uncertainty, particularly in countries with poor data quality or coverage.

Moreover, the inability to incorporate key structural variables, such as socioeconomic status, dental service coverage, or education, represents a substantial limitation that could influence the direction and magnitude of the observed associations. These factors are critical social determinants of oral health that could not be controlled in the models used.

Finally, the cross-sectional design limits the exploration of temporality and causal trajectories, preventing the evaluation of the dynamic evolution of tobacco exposure and its cumulative impact on periodontal health. Despite these limitations, the study provides a solid regional perspective on the association between smoking and periodontal disease, and its findings offer a useful basis for future individual research and for strengthening public health policies related to oral health.

## 6. Implications for Clinical Practice and Public Health

The results of this study have significant implications for clinical practice and public health in Latin America, particularly regarding the prevention and comprehensive management of periodontal diseases associated with tobacco use. The identification of associations across different age groups emphasizes the need to design interventions tailored to the epidemiological and cultural characteristics of each population, considering both tobacco use and the social determinants of health.

Smoking remains one of the primary modifiable risk factors for oral health. In this context, increasing awareness of the harmful effects of tobacco—both in active and passive smokers—should be a priority in prevention strategies. Clinical professionals should systematically integrate tobacco use history into the evaluation of patients with periodontal disease for both diagnostic and therapeutic purposes.

Furthermore, it is essential to strengthen public policies that limit environmental exposure to tobacco smoke, especially in enclosed spaces, to protect non-smokers. The implementation of smoking cessation programs within dental services can have a substantial impact, especially if directed not only at active smokers but also at those exposed passively. In summary, a holistic clinical and public health approach is needed, which considers the behavioral, environmental, and biological dimensions of smoking in periodontal disease.

## 7. Conclusions

This study highlighted a significant ecological-level association between periodontal disease and tobacco consumption, both active and passive, in men and women in Latin America. However, these associations should not be interpreted as causal or generalized to the individual level, given the ecological design of the study. Moreover, the variability in the statistical significance of the observed coefficients warrants cautious interpretation. Nonetheless, these results reinforce the role of tobacco as a relevant risk factor in oral health and underline the need to strengthen tobacco control policies, not only focused on reducing active consumption but also on effective protection from passive exposure.

Given the high prevalence of smoking in the region and the clinical implications of periodontal disease on overall health, it is imperative to implement comprehensive preventive strategies that include educational campaigns, regulation of smoke-free environments, and access to effective tobacco cessation programs. Future studies using individual-level data and longitudinal designs are needed to validate these findings, explore underlying biological mechanisms, and guide more precise interventions to reduce the burden of periodontal disease in high-vulnerability contexts.

## Figures and Tables

**Table 1:** Multiple Regression Models: Association Between Periodontal Disease Prevalence, Tobacco Use, and Age Group by Sex.

Sex	Tabaco Use	Number of Observations	Adjusted R^2^	F (gl)	*p* Value (Model)	Tobacco Coefficient (95% CI)	*p* Value (Tobacco)	Root MSE	Significant Predictor Association	Age Groups with Significant Association
Female	Active	60	0.372	12.65 (3.56)	<0.001	−84.58 (−331.39 to 162.24)	0.49	3690.8	No	55–85 (+) ≥85 (−)
Female	Passive	80	0.904	187.1 (4.75)	<0.001	29.29 (−54.25 to 112.82)	0.48	3192.8	No	21–54 (+) 55–85 (+) ≥85 (+)
Male	Active	60	0.460	17.76 (3.56)	<0.001	−4.53 (−210.24 to 201.18)	0.96	3872.5	No	55–85 (+)
Male	Passive	80	0.925	245.7 (4.75)	<0.001	93.38 (−0.60 to 186.82)	0.05	3261.7	Limit	21–54 (+) 55–85 (+) ≥85 (+)

## Data Availability

The data from this article will be made available by the authors on reasonable request.
